# Association between SII and hepatic steatosis and liver fibrosis: A population-based study

**DOI:** 10.3389/fimmu.2022.925690

**Published:** 2022-09-15

**Authors:** Ruijie Xie, Mengde Xiao, Lihong Li, Nengqian Ma, Mingjiang Liu, Xiongjie Huang, Qianlong Liu, Ya Zhang

**Affiliations:** ^1^ Department of Hand and Microsurgery, The Affiliated Nanhua Hospital, Hengyang Medical School, University of South China, Hengyang, China; ^2^ Department of Medical Records Management Center, The Affiliated Nanhua Hospital, Hengyang Medical School, University of South China, Hengyang, China; ^3^ Department of General Surgery, The Affiliated Nanhua Hospital, Hengyang Medical School, University of South China, Hengyang, China

**Keywords:** systemic immune-inflammatory index, NAFLD, NHANES, hepatic steatosis, liver fibrosis

## Abstract

**Background:**

The systemic immune-inflammation index (SII) is a novel marker of inflammation, and hepatic steatosis and fibrosis are associated with inflammation. This study aimed to investigate the possible relationship between SII and hepatic steatosis and fibrosis.

**Methods:**

The datasets from the National Health and Nutrition Examination Survey (NHANES) 2017–2020 were used in a cross-sectional investigation. Multivariate linear regression models were used to examine the linear connection between SII and controlled attenuation parameter (CAP) and liver stiffness measurement (LSM). Fitted smoothing curves and threshold effect analysis were used to describe the nonlinear relationship.

**Results:**

This population-based study included a total of 6,792 adults aged 18–80 years. In a multivariate linear regression analysis, a significant positive association between SII and CAP was shown [0.006 (0.001, 0.010)]. This positive association in a subgroup analysis was maintained in men [0.011 (0.004, 0.018)] but not in women. Furthermore, the association between SII and CAP was nonlinear; using a two-segment linear regression model, we found an inverted U-shaped relationship between SII and CAP with an inflection point of 687.059 (1,000 cells/µl). The results of the multiple regression analysis showed that the relationship between SII and LSM was not significant (P = 0.263).

**Conclusions:**

Our findings imply that increased SII levels are linked to hepatic steatosis, but SII is not linked to liver fibrosis. To confirm our findings, more large-scale prospective investigations are needed.

## Background

Non-alcoholic fatty liver disease (NAFLD) is the most prevalent chronic liver disease worldwide and one of the primary causes of severe liver disease (1–3). NAFLD is defined as excessive fat infiltration into the liver in the absence of substantial alcohol intake or secondary causes (4), which includes a variety of histological alterations in the liver, ranging from simple steatosis through leukocyte infiltration and hepatocyte ballooning to severe liver fibrosis and cirrhosis (5, 6). Transient elastography is widely used in the screening of NAFLD due to its good accuracy and noninvasive feature (7, 8); controlled attenuation parameter (CAP) and liver stiffness measurement (LSM) were used to assess hepatic steatosis and fibrosis, respectively (9, 10).

The systemic immune-inflammation index (SII) is an integrated and novel inflammatory biomarker as reported in the study by Hu et al. (11) in 2014, which could reflect the local immune response and systemic inflammation in the whole human body (12–15). SII has been used in past studies to predict and evaluate the prognosis of various solid tumors, such as gastric cancer (16, 17), non-small cell lung cancer (18, 19), pancreatic cancer (20), and esophageal cancer (21, 22). In addition, SII also has a high value for the prognosis of cardiovascular disease (23–27). Inflammation is a hallmark of NAFLD progression, and the recruitment of circulating inflammatory cells and the upregulation of inflammatory mediators play an important role in hepatic steatosis and fibrosis (28–31). Fontes-Cal et al. (32) reported that plasma cytokines and clinical parameters of inflammation could serve as a new strategy for monitoring NAFLD progression. However, the relationship between SII and hepatic steatosis and fibrosis remains unclear.

As a result, we examined the relationship between SII and CAP and LSM in adults in this study, utilizing a large sample of people aged 18 to 80 years from the National Health and Nutrition Examination Survey (NHANES).

## Methods

### Study population

**Figure 1:**
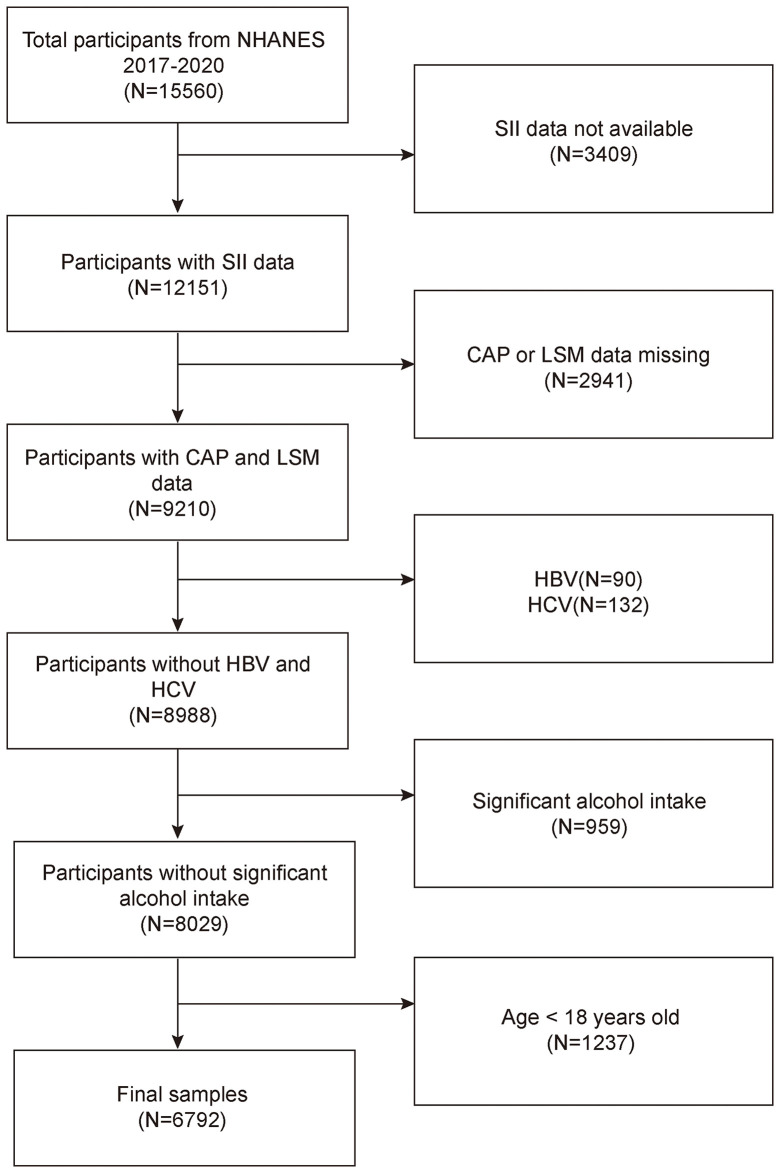
Flowchart of participant selection. NHANES, National Health and Nutrition Examination Survey; SII, systemic immune-inflammation index; CAP, controlled attenuation parameter; LSM, liver stiffness measurement.

The NHANES is a representative survey of the US national population that uses a complicated, multistage, and probabilistic sampling methodology to provide a wealth of information about the general US population’s nutrition and health (33). The 2017–2020 continuous cycle of the US NHANES dataset was used for this investigation. We excluded 3,409 participants with missing SII data, 2,941 with missing CAP or LSM data, 90 hepatitis B antigen-positive and 132 hepatitis C antibody-positive or hepatitis C RNA-positive samples, 959 participants with significant alcohol consumption (ever have 4, 5, or more drinks every day), and 1,237 participants younger than 18 years from the 15,560 eligible individuals. The study eventually included 6,792 participants. **Figure 1** illustrates the sample selection flowchart.

### Study variables

The dependent variable in this study is the systemic immune-inflammation index, with CAP and LSM as the intended independent variables. In our analysis, SII was designed as an exposure variable. Lymphocyte, neutrophil, and platelet counts were measured by complete blood count using automated hematology analyzing devices (Coulter^®^DxH 800 analyzer) and presented as ×10^3^ cells/ml. SII as an exposure variable was derived from platelet count × neutrophil count/lymphocyte count (11, 13, 34). CAP and LSM were designed as outcome variables to measure hepatic steatosis and liver fibrosis. The NHANES staff evaluated participants for Vibration controlled transient elastography (VCTE) using the FibroScan^®^-equipped model 502 V2 Touch. According to a recent landmark study, CAP values, also known as CAP, ≥274 dB/m was considered indicative of NAFLD status because of 90% sensitivity in detecting all degrees of hepatic steatosis (9). Based on two past studies, CAP ≥302 dB/m was defined in this study as having severe steatosis at the base of NAFLD (4, 35). Fibrosis grade was determined by liver stiffness with cutoff values of 8.2, 9.7, and 13.6 kPa for fibrosis grades ≥F2, ≥F3, and F4, respectively, and was optimized using the Jorden index (36, 37). Covariates included age, gender, race, Body Mass Index (BMI), education level, family income-to-poverty ratio, activity status, alanine transaminase (ALT), weight, alkaline phosphatase (ALP), waist circumference, aspartate aminotransferase (AST), total calcium, total cholesterol, direct High-Density Lipoprotein Cholesterol (HDL-C), Low-Density Lipoprotein Cholesterol (LDL-C), triglyceride, serum phosphorus, and smoking status.

### Statistical analysis

The statistical study was carried out using the statistical computing and graphics software R (version 4.1.3) and EmpowerStats (version:2.0). Baseline tables for the study population were statistically described by CAP and LSM subgroups; continuous variables are described using mean values plus or minus standard deviation (SD) and weighted linear regression models. The beta values and 95% confidence intervals were calculated using multivariate linear regression analysis between the SII and CAP and LSM. The multivariate test was built using three models: model 1: no variables adjusted; model 2: gender, age, and race adjusted; model 3: adjusted for all covariates. By adjusting the variables, smoothed curve fits were done simultaneously. A threshold effects analysis model was used to examine the relationship and inflection point between SII and CAP. Finally, the same statistical study methods described above were conducted for the gender subgroups. It was determined that P < 0.05 was statistically significant. We used a weighting approach to reduce the significant volatility of our dataset.

## Results

### Baseline characteristics

In this study, 6,792 adults were included based on the inclusion and exclusion criteria, and the average age of the participants was 48.58 ± 18.50 years. Among these participants, 45.39% were men, 54.61% were women, 33.82% were non-Hispanic white, 25.28% were non-Hispanic black, and 12.38% were Mexican American, and 28.52% were from other races. The mean (SD) concentrations of CAP, LSM, and SII were 262.49 (62.84) dB/m, 5.84 (4.81) kPa, and 515.48 (341.66) (1,000 cells/µl), respectively.

**Table 1** lists all clinical characteristics of the participants with CAP as a column-stratified variable. In comparison to the non-NAFLD group, the severe steatosis group is more likely to be men and older, with a higher proportion of non-Hispanic blacks and Mexican Americans; with higher smoking status; and higher levels of BMI, waist circumference, AST, ALT, ALP, total cholesterol, LDL cholesterol, triglyceride, LSM, and SII but lower levels of direct HDL cholesterol and serum phosphorus.

**Table 1:** Weighted characteristics of the study population based on controlled attenuated parameter (CAP).

	Non-NAFLD (CAP < 274, n = 3,901)	NAFLD (274 ≤ CAP < 302, n = 1,031)	Severe steatosis (CAP ≥ 302, n = 1,860)	P value
Age (years)	46.135 ± 19.305	51.607 ± 17.552	52.013 ± 16.389	<0.001
*Gender (%)*				<0.001
Men	41.989	43.938	53.333	
Women	58.011	56.062	46.667	
*Race/Ethnicity (%)*				<0.001
Non-Hispanic White	33.017	32.590	36.183	
Non- Hispanic Black	28.198	24.151	19.785	
Mexican American	9.510	14.646	17.151	
Other Race	29.275	28.613	26.882	
*Education lever (%)*				0.089
Less than high school	16.597	18.924	18.932	
High school	22.555	22.809	23.720	
More than high school	60.848	58.267	57.348	
*Moderate activities (%)*				<0.001
Yes	46.091	40.543	37.312	
No	53.909	59.457	62.688	
Smoked at least 100 cigarettes				<0.001
Yes	32.479	31.620	40.430	
No	67.521	68.380	59.570	
Income to poverty ratio	2.646 ± 1.663	2.716 ± 1.601	2.649 ± 1.608	0.520
BMI (kg/m^2^)	26.902 ± 5.946	31.480 ± 6.801	34.873 ± 7.725	<0.001
Waist circumference (cm)	92.211 ± 14.260	104.038 ± 13.584	112.974 ± 15.613	<0.001
Laboratory features				
Total calcium (mmol/L)	2.320 ± 0.092	2.320 ± 0.098	2.318 ± 0.097	0.673
Total cholesterol (mmol/L)	4.714 ± 1.024	4.890 ± 1.045	4.848 ± 1.054	<0.001
Triglyceride(mmol/L)	0.979 ± 0.727	1.379 ± 1.552	1.629 ± 1.193	<0.001
LDL- cholesterol(mmol/L)	2.750 ± 0.891	2.903 ± 0.932	2.868 ± 0.927	<0.001
HDL- cholesterol(mmol/L)	1.474 ± 0.400	1.336 ± 0.392	1.211 ± 0.342	<0.001
ALT (IU/L)	18.311 ± 13.825	22.347 ± 15.461	27.978 ± 20.136	<0.001
AST (IU/L)	20.302 ± 10.862	21.414 ± 12.840	23.287 ± 14.532	<0.001
ALP(IU/L)	74.800 ± 24.197	78.789 ± 22.622	81.974 ± 25.317	<0.001
Serum phosphorus (mmol/L)	1.162 ± 0.166	1.149 ± 0.164	1.137 ± 0.169	<0.001
LSM (kPa)	5.122 ± 3.873	5.895 ± 4.414	7.328 ± 6.229	<0.001
SII (1,000 cells/µl)	509.876 ± 364.567	504.868 ±289.827	533.097 ± 317.184	0.030

Mean ± SD for continuous variables: P value was calculated by weighted linear regression model.

% for categorical variables: P value was calculated by weighted chi-square test. BMI, body mass index; LDL- cholesterol, low-Density Lipoprotein Cholesterol; HDL- cholesterol, high-Density Lipoprotein Cholesterol; ALT, alanine transaminase; ALP, alkaline phosphatase; AST, aspartate aminotransferase; LSM, liver stiffness measure ; CAP, controlled attenuation parameter; SII, systemic immune-inflammation index.

**Table 2** lists all clinical features of the individuals with LSM as a column-stratified variable. In comparison to the normal group, the cirrhosis group is more likely to be men and older, with a higher proportion of non-Hispanic blacks and Mexican Americans; with higher smoking status; and higher levels of BMI, waist circumference, AST, ALT, ALP, LDL cholesterol, triglyceride, CAP, and SII but lower levels of HDL cholesterol, total cholesterol, and serum phosphorus.

**Table 2:** Weighted characteristics of the study population based on median liver stiffness measurement (LSM).

	Normal group(LSM<8.2, n = 6,098)	Significant fibrosis(8.0≤LSM<9.7, n = 283)	Advanced fibrosis(9.7≤LSM<13.6, n = 223)	Cirrhosis(LSM≥13.6, n = 188)	P value
Age (years)	47.859 ± 18.578	53.484 ± 16.578	56.309 ± 16.466	55.250 ± 16.525	<0.001
*Gender (%)*					<0.001
Men	44.654	49.823	49.776	57.447	
Women	55.346	50.177	50.224	42.553	
*Race/Ethnicity (%)*					0.018
Non-Hispanic White	33.585	31.449	38.565	39.362	
Non- Hispanic Black	25.057	32.509	24.215	22.872	
Mexican American	12.283	12.014	12.108	16.489	
Other Race	29.075	24.028	25.112	21.277	
*Education lever (%)*					0.898
Less than high school	17.405	19.343	18.894	20.330	
High school	22.668	24.818	26.267	24.176	
More than high school	59.927	55.839	54.839	55.495	
*Moderate activities (%)*					0.003
Yes	43.736	36.396	33.632	34.574	
No	56.264	63.604	66.368	65.426	
Smoked at least 100 cigarettes					0.081
Yes	33.864	36.749	44.843	40.426	
No	66.136	63.251	55.157	59.574	
Income to poverty ratio	2.669 ± 1.649	2.642 ± 1.580	2.504 ± 1.511	2.482 ± 1.514	0.270
BMI (kg/m^2^)	29.022 ± 6.691	33.734 ± 9.207	38.043 ± 9.651	38.538 ± 11.549	<0.001
Waist circumference (cm)	97.955 ± 15.952	109.169 ± 19.514	119.096 ± 16.628	120.849 ± 20.775	<0.001
Laboratory features					
Total calcium (mmol/L)	2.320 ± 0.093	2.321 ± 0.106	2.313 ± 0.109	2.307 ± 0.100	0.219
Total cholesterol (mmol/L)	4.792 ± 1.027	4.666 ± 1.104	4.720 ± 1.156	4.537 ± 1.115	0.002
Triglyceride(mmol/L)	1.194 ± 1.027	1.389 ± 1.353	1.597 ± 1.708	1.353 ± 0.902	<0.001
LDL- cholesterol(mmol/L)	2.819 ± 0.897	2.733 ± 0.999	2.752 ± 1.067	2.547 ± 0.940	0.025
HDL- cholesterol(mmol/L)	1.395 ± 0.397	1.276 ± 0.351	1.247 ± 0.428	1.238 ± 0.449	<0.001
ALT (IU/L)	20.657 ± 14.773	26.575 ± 19.780	30.677 ± 27.380	33.253 ± 32.692	<0.001
AST (IU/L)	20.553 ± 9.249	24.085 ± 16.192	26.926 ± 19.327	34.086 ± 40.412	<0.001
ALP(IU/L)	76.325 ± 23.230	82.905 ± 26.756	85.604 ± 30.118	93.269 ± 39.714	<0.001
Serum phosphorus (mmol/L)	1.155 ± 0.166	1.148 ± 0.174	1.131 ± 0.174	1.131 ± 0.171	0.045
CAP (dB/m)	257.063 ± 60.343	297.473 ± 62.943	316.704 ± 61.176	321.665 ± 66.575	<0.001
SII (1,000 cells/µl)	513.208 ± 335.813	537.926 ± 463.726	519.919 ± 322.459	549.945 ± 335.262	0.328

Mean ± SD for continuous variables: P value was calculated by weighted linear regression model.

% for categorical variables: P value was calculated by weighted chi-square test. BMI, body mass index; LDL- cholesterol, low-Density Lipoprotein Cholesterol; HDL- cholesterol, high-Density Lipoprotein Cholesterol; ALT, alanine transaminase; ALP, alkaline phosphatase; AST, aspartate aminotransferase; LSM, liver stiffness measure ; CAP, controlled attenuation parameter; SII, systemic immune-inflammation index.

### Association between systemic immune-inflammation index (SII) and controlled attenuation parameter (CAP)

**Table 3** showed the results of the multivariate regression analysis. In the unadjusted model [0.006 (0.001, 0.010)], SII was highly associated with CAP. However, after adjusting for gender, age, and race variables, this significant positive correlation became insignificant in model 2 [0.002 (-0.002, 0.007)]. After adjusting for all covariates, the relationship between SII and CAP became negative in model 3 [-0.002 (-0.009, 0.004)].

**Table 3:** The association between SII and CAP.

	Model 1 β (95% CI) P value	Model 2 β (95% CI) P value	Model 3 β (95% CI) P value
CAP (dB/m)	0.006 (0.001, 0.010)	0.002 (-0.002, 0.007)	-0.002 (-0.009, 0.004)
	0.011	0.285	0.443
*Stratified by CAP*			
Non-NAFLD	Reference	Reference	Reference
NAFLD	0.004 (0.000, 0.009)	0.005 (0.000, 0.009)	-0.003 (-0.010, 0.003)
	0.041	0.030	0.335
Severe steatosis	0.003 (0.001, 0.008)	0.005 (0.001, 0.010)	-0.002 (-0.008, 0.002)
	0.012	0.026	0.518
*Stratified by gender*			
Men	0.011 (0.004, 0.018)	0.004 (-0.004, 0.011)	-0.003 (-0.013, 0.007)
	0.003	0.325	0.596
Women	0.003 (-0.003, 0.008)	0.002 (-0.003, 0.007)	-0.003 (-0.011, 0.005)
	0.335	0.470	0.491

Model 1: no covariates were adjusted. Model 2: age, gender, and race were adjusted. Model 3: age, gender, race, educational level, BMI, family income-to-poverty ratio, moderate activities, smoking status, ALP, ALT, AST, total calcium, total cholesterol, triglyceride, LDL, HDL-C, waist circumference, and serum phosphorus were adjusted.

In the subgroup analysis stratified by gender and race, the model is not adjusted for sex and race, respectively. NAFLD, non-alcoholic fatty liver disease; CAP, controlled attenuation parameter; SII, systemic immune-inflammation index.

In subgroup analyses stratified by gender, our results suggest that the positive association between SII and CAP is independently significantly positive in men [0.011 (0.004, 0.018)] but not statistically significant in all models for women. When we performed a subgroup analysis stratified by the degree of hepatic steatosis, the SII showed a strong positive correlation with both the NAFLD group and the severe steatosis group in both the unadjusted and partially adjusted models using the non-NAFLD group as the reference group.

**Figure 2:**
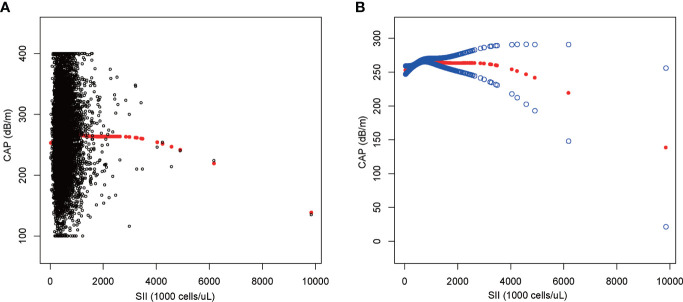
The association between SII and CAP. **(A)** Each black point represents a sample. **(B)** The solid red line represents the smooth curve fit between variables. Blue bands represent the 95% confidence interval from the fit. SII, systemic immune-inflammation index; CAP, controlled attenuation parameter.

**Figure 3:**
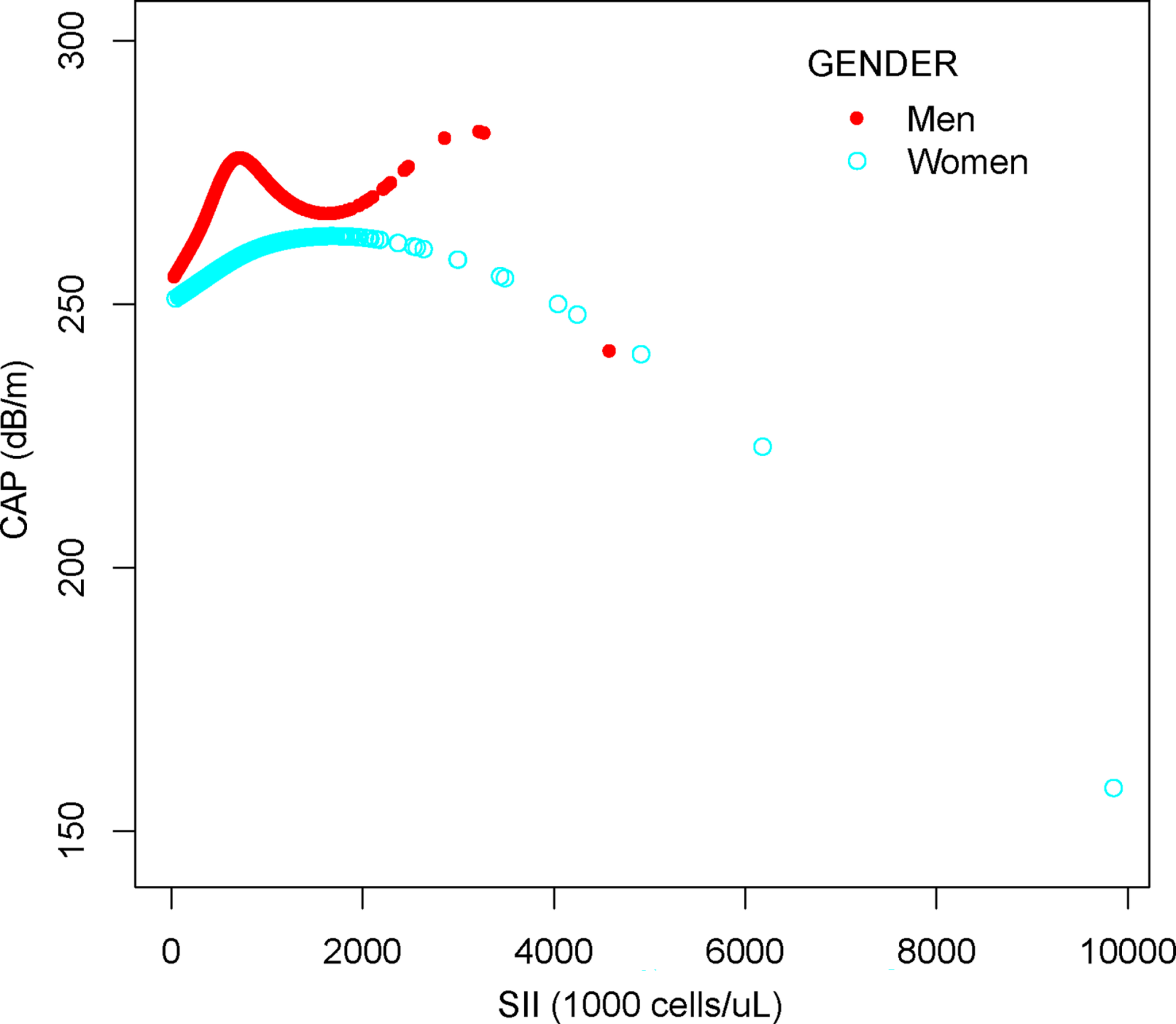
The association between SII and CAP stratified by gender. SII, systemic immune-inflammation index; CAP, controlled attenuation parameter.

We performed a smooth curve fit to describe the nonlinear relationship between SII and CAP (**Figures 2**, **3**). Using a two-segment linear regression model, we found an inverted U-shaped relationship between SII and CAP with an inflection point of 687.059 (1,000 cells/µl). After stratifying the analysis by gender, an inverted U-shaped curve was also present in men and women, with inflection points of 591.000 (1,000 cells/µl) and 749.692 (1,000 cells/µl), respectively (**Table 4**).

**Table 4:** Threshold effect analysis of SII on CAP using two-piecewise linear regression model.

CAP (dB/m)	Adjusted β (95% CI)P value
*SII*	
Inflection point	687.059
SII<687.059 (1,000 cells/µl)	0.026 (0.017, 0.035) <0.0001
SII>687.059 (1,000 cells/µl)	-0.008 (-0.015, -0.002) 0.0107
Log likelihood ratio	<0.001
*Men*	
Inflection point	591.000
SII<591.000 (1,000 cells/µl)	0.045 (0.029, 0.062) <0.0001
SII>591.000 (1,000 cells/µl)	-0.008 (-0.019, 0.003) 0.1556
Log likelihood ratio	<0.001
*Women*	
Inflection point	749.692
SII<749.692 (1,000 cells/µl)	0.022 (0.011, 0.033) 0.0001
SII>749.692 (1,000 cells/µl)	-0.008 (-0.016, -0.000) 0.0372
Log likelihood ratio	<0.001

Age, gender, race, educational level, BMI, family income-to-poverty ratio, moderate activities, smoking status, ALP, ALT, AST, total calcium, total cholesterol, triglyceride, LDL, HDL-C, waist circumference, and serum phosphorus were adjusted. LSM, liver stiffness measure ; SII, systemic immune-inflammation index.

### Association between SII and LSM

**Figure 4:**
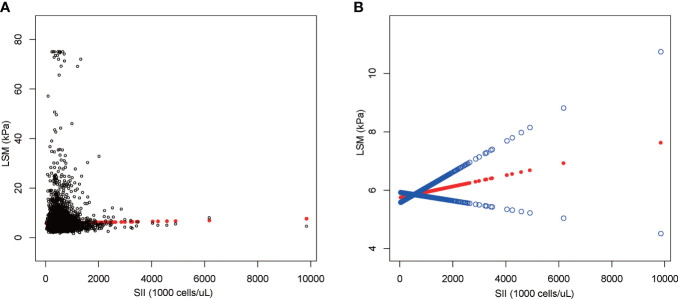
The association between SII and LSM. **(A)** Each black point represents a sample. **(B)** The solid red line represents the smooth curve fit between variables. Blue bands represent the 95% confidence interval from the fit. SII, systemic immune-inflammation index; LSM, liver stiffness measurement.

The results of multiple regression analysis showed a positive but insignificant correlation between SII and LSM (**Table 5**). Moreover, the effect value was shown to be zero within three decimal places because the units of SII were too small [0.000 (-0.000, 0.001)]. Among all subgroup analyses, SII showed a statistically significant negative correlation with LSM only in the significant fibrosis group [-0.000 (-0.000, -0.000), P = 0.044]. The nonlinear relationship was characterized by smooth curve fittings (**Figure 4**).

**Table 5:** The association between SII and LSM.

	Model 1 β (95% CI) P value	Model 2 β (95% CI) P value	Model 3 β (95% CI) P value
LSM (kPa)	0.000 (-0.000, 0.001)	0.000 (-0.000, 0.001)	-0.000 (-0.001, 0.001)
	0.263	0.182	0.927
*Stratified by LSM*			
Normal group	Reference	Reference	Reference
Significant fibrosis	-0.000 (-0.000, -0.000)	-0.000 (-0.000, -0.000)	-0.000 (-0.000, -0.000)
	0.044	0.040	0.513
Advanced fibrosis	-0.000 (-0.000, -0.000)	-0.000 (-0.000, -0.000)	0.000 (-0.000, 0.001)
	0.920	0.967	0.357
Cirrhosis	-0.001 (-0.008, 0.006)	-0.000 (-0.008, 0.007)	0.000 (-0.013, 0.013)
	0.813	0.924	0.967
*Stratified by gender*			
Men	0.000 (-0.000, 0.001)	0.000 (-0.001, 0.001)	-0.000 (-0.001, 0.001)
	0.432	0.867	0.872
Women	0.000 (-0.000, 0.001)	0.000 (-0.001, 0.001)	-0.000 (-0.001, 0.001)
	0.145	0.095	0.970

Model 1: no covariates were adjusted. Model 2: age, gender, and race were adjusted. Model 3: age, gender, race, educational level, BMI, family income-to-poverty ratio, moderate activities, smoking status, ALP, ALT, AST, total calcium, total cholesterol, triglyceride, LDL, HDL-C, waist circumference, and serum phosphorus were adjusted.

In the subgroup analysis stratified by gender and race, the model is not adjusted for sex and race, respectively. CAP, controlled attenuation parameter; SII, systemic immune-inflammation index.

## Discussion

In our study sample, which is nationally representative of US adults, SII levels were positively correlated with hepatic steatosis and there was no significant correlation between SII levels and liver fibrosis. Notably, we found an inverted U-shaped association between SII and CAP, with an inflection point of 687.059 (1,000 cells/µl). This indicated that SII was an independent crisis factor for hepatic steatosis when the SII was less than 687.059 (1,000 cells/µl).

To our knowledge, this is the first study to investigate SII with hepatic steatosis and fibrosis. In previous studies on the liver, SII has often been used as a predictor of prognostic survival in patients with hepatocellular carcinoma or intrahepatic cholangiocarcinoma (ICC) (38–40). Ren et al. (41) reported that among 28 patients with ICC who received liver transplantation, the 1-, 3-, and 5-year survival rates were significantly lower in the high-SII group than those in the low-SII group, and that SII could be used to predict survival in patients with ICC who received liver transplantation. Similarly, another study from China showed that SII was a valid prognostic factor for predicting the prognosis of patients undergoing radical hepatectomy for ICC, while neutrophil-to-lymphocyte ratio (NLR), platelet-to-lymphocyte ratio (PLR), and lymphocyte-to-monocyte ratio (LMR) were not associated with clinical outcomes in these patients (42).

At present, many epidemiological studies have proven that inflammation is related to the progression of NAFLD (43–45). A large multicenter cohort of NAFLD patients from Italy and Finland showed that steatosis, ballooning, and lobular inflammation were independently associated with significant fibrosis. In addition, the authors found that a third of patients with significant fibrosis did not have non-alcoholic steatohepatitis (NASH) when they analyzed biopsy specimens taken from NAFLD patients at a single time point, a result that far exceeded expectations (46). The lack of significant association between SII and LSM found in our results may explain this phenomenon. Haukeland et al. (28) evaluated serum samples from 47 histologically validated NAFLD patients and showed that NAFLD patients are characterized by low-grade systemic inflammation. High chemokine (C-C motif) ligand 2 (CCL2)/monocyte chemoattractant protein 1 (MCP1) levels in NASH may be important for the transition from simple steatosis to NASH (28). Our results demonstrate a significant positive relationship between SII and CAP; in other words, inflammation has a strong positive correlation with hepatic steatosis. Not only that, but the positive association between SII and CAP differs significantly by gender. Men with NAFLD have more severe hepatic steatosis than women, and postmenopausal women have greater hepatic steatosis than premenopausal women, according to several studies, suggesting that the gender difference in NAFLD is related to sex hormones (47, 48). Furthermore, a recent experimental animal study found that Formyl Peptide Receptor 2 (FPR2) expression is higher in female mice than that in male mice, making females more resistant to the development and progression of NAFLD, and the severe damage seen in FPR2-depleted females supports FPR2’s protective role in female mice’s liver (49). In addition to sex, race, age, and other covariates may also be factors influencing the relationship between SII and CAP, and multiple factors interacting with each other may also be the reason why the relationship between SII and CAP in this study was not significant in model 2 and model 3.

NAFLD includes a disease continuum from steatosis with or without mild inflammation to NASH, characterized by necrotizing inflammation and faster fibrotic progression than NAFLD (50). The mechanisms behind the connection between inflammation and NAFLD progression are unclear. One theory is that nutrient overload is the primary cause of NAFLD, with excess visceral fat causing macrophage infiltration into tissue compartments, resulting in a pro-inflammatory state that increases insulin resistance. Inappropriate lipolysis in the presence of insulin resistance causes aberrant fatty acid transport to the liver, resulting in a decrease in metabolic capacity. Lipotoxic lipids are formed as a result of lipid metabolic imbalances, which cause cellular stress, inflammasome activation, and apoptotic cell death, as well as stimulation of inflammation, tissue regeneration, and fibrogenesis (51, 52). This may be the mechanism leading to the progression of hepatic steatosis and fibrosis (53). Another theory is that metabolic imbalance and inflammation in NAFLD are caused by the liver’s interdependence and interaction with other organs (54–56). For example, differences in gut microbiota composition have been observed in NAFLD patients compared to the general population, and some data suggest the presence of fecal microbiome signatures associated with advanced fibrosis (57). Furthermore, substances produced by bacteria or bile acid metabolism can influence liver inflammation and disease progression in NAFLD, although a clear causal relationship has not been established (50, 57).

Our study has some limitations. First, this is a cross-sectional analysis; thus, temporality cannot be ascertained. Furthermore, despite adjusting several relevant confounders, we were unable to rule out the impact of additional confounding factors; therefore, our findings should be interpreted with caution. Third, due to the limitations of the NHANES database, the covariates of this study did not include participants’ medications use, and anti-inflammatory medications are often used in patients with NAFLD; therefore, our findings may not fully reflect the true situation. Fourth, the degree of hepatic steatosis and liver fibrosis in this study was judged by transient elastography, and although several studies have demonstrated the extremely high accuracy of transient elastography (58–60), it still cannot be the same as biopsy; therefore, our results may not be the same as using biopsy as a judgment of hepatic steatosis and liver fibrosis. Despite these limitations, our study has several advantages. Because we used a nationally representative sample, our study is representative of a multiethnic and gender-diverse population of adults in the United States. In addition to this, the large sample size included in our study allowed us to perform a subgroup analysis.

## Conclusion

Our findings imply that increased SII levels are linked to hepatic steatosis, but SII is not linked to liver fibrosis. To confirm our findings, more large-scale prospective investigations are needed.

## Data availability statement

Publicly available datasets were analyzed in this study. This data can be found here: www.cdc.gov/nchs/nhanes/.

## Ethics statement

This study was reviewed and approved by NCHS Ethics Review Board. The patients/participants provided their written informed consent to participate in this study.

## Author contributions

RX and YZ designed the research. RX, YZ, MX and LL collected and analyzed the data. RX, XH and YZ drafted the manuscript. ML, NM and RX revised the manuscript. All authors contributed to the article and approved the submitted version.

## Conflict of interest

The authors declare that the research was conducted in the absence of any commercial or financial relationships that could be construed as a potential conflict of interest.

## Publisher’s note

All claims expressed in this article are solely those of the authors and do not necessarily represent those of their affiliated organizations, or those of the publisher, the editors and the reviewers. Any product that may be evaluated in this article, or claim that may be made by its manufacturer, is not guaranteed or endorsed by the publisher.

## Abbreviations

NAFLD, non-alcoholic fatty liver disease; CAP, controlled attenuation parameter; LSM, liver stiffness measurement; SII, systemic immune-inflammation index; NHANES, National Health and Nutrition Examination Survey; ICC, intrahepatic cholangiocarcinoma; NLR, neutrophil-to-lymphocyte ratio; PLR, platelet-to-lymphocyte ratio; LMR, lymphocyte-to-monocyte ratio; NASH, non-alcoholic steatohepatitis.
