# Supplementary material for: Association between SII and hepatic steatosis and liver fibrosis: A population-based study
Source: Front Immunol. 2022 Sep 15;13:925690. doi: 10.3389/fimmu.2022.925690 (PMC9520084; doi:10.3389/fimmu.2022.925690)
Supplement: Supplementary file 1 [file Table_1.doc]

| **SII [(1000 cells/uL)](https://wwwn.cdc.gov/Nchs/Nhanes/2017-2018/P_CBC.htm#LBDLYMNO)** | **Q1** | **Q2** | **Q3** | **Q4** | ***P* value** |
| --- | --- | --- | --- | --- | --- |
|  | **(n=1695)** | **(n=1700)** | **(n=1699)** | **(n=1698)** |  |
| Age (years) | 48.326 ± 17.965 | 47.713 ± 18.519 | 48.529 ± 18.593 | 49.735 ± 18.876 | 0.014 |
| *Sex, n (%)* |  |  |  |  | < 0.001 |
| Male | 50.678 | 48.647 | 42.496 | 39.753 |  |
| Female | 49.322 | 51.353 | 57.504 | 60.247 |  |
| *Race/Ethnicity (%)* |  |  |  |  | < 0.001 |
| Non-Hispanic White | 21.475 | 33.706 | 37.552 | 42.521 |  |
| Non-Hispanic Black | 39.115 | 23.529 | 20.365 | 18.139 |  |
| Mexican American | 10.737 | 13.941 | 12.890 | 11.955 |  |
| Other race/ethnicity | 28.673 | 28.824 | 29.194 | 27.385 |  |
| *Education level (%)* |  |  |  |  | 0.747 |
| Less than high school | 16.667 | 18.410 | 17.928 | 17.483 |  |
| High school | 22.637 | 21.979 | 22.581 | 24.489 |  |
| More than high school | 60.696 | 59.611 | 59.491 | 58.028 |  |
| Body mass index (kg/m2) | 28.640 ± 6.429 | 29.237 ± 6.934 | 30.140 ± 7.654 | 31.092 ± 8.517 | < 0.001 |
| Waist circumference (cm) | 96.867 ± 16.049 | 98.608 ± 16.284 | 100.319 ± 17.091 | 103.042 ± 18.608 | < 0.001 |
| Income to poverty ratio | 2.677 ± 1.660 | 2.731 ± 1.648 | 2.672 ± 1.650 | 2.549 ± 1.591 | 0.021 |
| *Moderate activities (%)* |  |  |  |  | 0.029 |
| Yes | 45.664 | 43.706 | 42.025 | 39.988 |  |
| No | 54.336 | 56.294 | 57.975 | 60.012 |  |
| *Smoked at least 100 cigarettes in life, n (%)* |  |  |  |  | 0.027 |
| Yes | 31.032 | 35.118 | 35.550 | 36.396 |  |
| No | 68.968 | 64.882 | 64.450 | 64.604 |  |
| Laboratory features |  |  |  |  | < 0.001 |
| Total calcium (mmol/L) | 2.321 ± 0.094 | 2.321 ± 0.090 | 2.318 ± 0.093 | 2.320 ± 0.100 | 0.851 |
| Total cholesterol (mmol/L) | 4.796 ± 1.041 | 4.793 ± 1.063 | 4.796 ± 1.025 | 4.725 ± 1.024 | 0.126 |
| Triglyceride(mmol/L) | 1.150 ± 1.309 | 1.245 ± 1.121 | 1.278 ± 0.998 | 1.207 ± 0.740 | 0.089 |
| LDL- cholesterol(mmol/L) | 2.794 ± 0.886 | 2.841 ± 0.920 | 2.820 ± 0.938 | 2.766 ± 0.892 | 0.379 |
| HDL- cholesterol(mmol/L) | 1.410 ± 0.402 | 1.376 ± 0.405 | 1.363 ± 0.399 | 1.375 ± 0.394 | 0.005 |
| ALT (IU/L) | 21.890 ± 15.766 | 22.542 ± 16.001 | 21.398 ± 15.408 | 20.516 ± 18.865 | 0.004 |
| AST (IU/L) | 22.585 ± 14.083 | 21.711 ± 11.604 | 20.827 ± 11.325 | 20.044 ± 12.031 | < 0.001 |
| ALP(IU/L) | 75.009 ± 24.790 | 75.954 ± 23.449 | 77.206 ± 22.792 | 81.362 ± 26.287 | < 0.001 |
| Serum phosphorus (mmol/L) | 1.160 ± 0.169 | 1.153 ± 0.163 | 1.152 ± 0.164 | 1.147 ± 0.171 | 0.176 |
| SII [(1000 cells/uL)](https://wwwn.cdc.gov/Nchs/Nhanes/2017-2018/P_CBC.htm#LBDLYMNO) | 236.194 ± 58.935 | 380.623 ± 38.202 | 527.623 ± 51.685 | 917.118 ± 449.264 | < 0.001 |
| LSM (kPa) | 5.741 ± 4.342 | 5.822 ± 4.548 | 5.802 ± 5.270 | 6.009 ± 5.006 | 0.403 |
| CAP (dB/m) | 257.834 ± 62.039 | 259.899 ± 61.819 | 265.353 ± 63.117 | 266.881 ± 63.976 | < 0.001 |

**Table1:** Weighted characteristics of the study population based on SII quartiles.

Mean±SD for continuous variables: the *P* value was calculated by the weighted linear regression model.

(%) for categorical variables: the *P* value was calculated by the weighted chi-square test.

Abbreviation: SII, systemic immune-inflammatory index.
